# Light-degradable hydrogels as dynamic triggers for gastrointestinal applications

**DOI:** 10.1126/sciadv.aay0065

**Published:** 2020-01-17

**Authors:** Ritu Raman, Tiffany Hua, Declan Gwynne, Joy Collins, Siddartha Tamang, Jianlin Zhou, Tina Esfandiary, Vance Soares, Simo Pajovic, Alison Hayward, Robert Langer, Giovanni Traverso

**Affiliations:** 1 The David H. Koch Institute for Integrative Cancer Research, Massachusetts Institute of Technology, Cambridge, MA 02139, USA.; 2 Division of Comparative Medicine, Massachusetts Institute of Technology, Cambridge, MA 02139, USA.; 3 Department of Chemical Engineering, Massachusetts Institute of Technology, Cambridge, MA 02139, USA.; 4 Department of Mechanical Engineering, Massachusetts Institute of Technology, Cambridge, MA 02139, USA.; 5 Division of Gastroenterology, Department of Medicine, Brigham and Women’s Hospital, Harvard Medical School, Boston, MA 02115, USA.

## Abstract

Triggerable materials capable of being degraded by selective stimuli stand to transform our capacity to precisely control biomedical device activity and performance while reducing the need for invasive interventions. Here, we describe the development of a modular and tunable light-triggerable hydrogel system capable of interfacing with implantable devices. We apply these materials to two applications in the gastrointestinal (GI) tract: a bariatric balloon and an esophageal stent. We demonstrate biocompatibility and on-demand triggering of the material in vitro, ex vivo, and in vivo. Moreover, we characterize performance of the system in a porcine large animal model with an accompanying ingestible LED. Light-triggerable hydrogels have the potential to be applied broadly throughout the GI tract and other anatomic areas. By demonstrating the first use of light-degradable hydrogels in vivo, we provide biomedical engineers and clinicians with a previously unavailable, safe, dynamically deliverable, and precise tool to design dynamically actuated implantable devices.

## Teaser

Light-triggerable hydrogels enable on-demand degradation of gastrointestinal-resident devices, reducing the need for invasive interventions.

## INTRODUCTION

Hydrogels are compelling building blocks for implantable devices, as their compliant, biocompatible, and absorbent properties closely match the characteristics of biological tissue (*1*–*3*). Recent advances in developing tough and stretchable hydrogels, from ionically or covalently cross-linked and interpenetrating polymer networks, have made it more feasible to deploy these materials in dynamic in vivo environments (*4*–*7*). Robust hydrogels have been targeted at a wide variety of in vivo applications, ranging from tissue engineering replacements for cartilage to implantable microdevices for drug delivery (*8*–*10*). Applications that require triggerable actuation or degradation of these hydrogel-based devices have largely relied on heat-, pH-, magnet-, or chemical-based stimuli to elicit material response (*2*, *11*, *12*). Examples include thermo-magnetically responsive micro-grippers for tissue biopsies (*13*), pH-actuated gastric-resident platforms (*14*, *15*), and chemically dissolvable devices for drug delivery and in vivo monitoring (*16*, *17*). These stimuli, while well suited for use in some target applications, come with a range of disadvantages that render them inappropriate for use in many in vivo applications. Heat stimuli can have off-target negative effects on surrounding tissue, magnetic stimuli can interfere with standard medical imaging technologies, pH stimuli are spatially restricted in the body to regions that match the material’s pH-responsive range, and chemical stimuli require contact with the material and are difficult to spatially control and deploy inside the body. There is thus a critical need for dynamically deliverable, biocompatible, spatially controllable, and noncontact stimuli that can trigger actuation and dissolution of a hydrogel in vivo. Optical stimuli match these requirements, motivating the design and implementation of a light-triggerable hydrogel that can be used as a dynamic trigger for a range of implantable devices. Such hydrogels have applicability throughout the body but are of especial interest in the gastrointestinal (GI) tract where the specific environmental variation and limits of pH and temperature can turn pH-, chemical-, and heat-based triggers into potentially toxic stimuli. Broad variation in body morphology and girth also limits the feasibility of external magnetic control. The GI tract, moreover, enables facile and temporary insertion of optical stimuli in the form of ingestible or endoscopically delivered light sources, making light a particularly useful trigger in this organ system. In other organ systems, where implants are embedded more deeply within tissues, noninvasive activation of a light trigger would require a device with an on-board light-emitting thin film or optical fiber. A light-based trigger thus offers a unique dynamically tunable functionality not generally found but critically needed in implantable GI devices.

Light-triggerable functionalities have been incorporated into polymeric backbones using azobenzene-, spirobenzopyran-, coumarin-, and nitrobenzyl-based moieties (*18*–*22*). Of these structures, nitrobenzyl-derived functional groups have been the most well suited to in vivo applications, based on the biocompatibility of the polymer’s degradation by-products following light-triggered cleavage (*23*–*25*). A substantial body of work has shown that poly(ethylene glycol) (PEG) hydrogel networks polymerized using acrylated *ortho*-nitrobenzyl (oNB) can be triggered on demand by blue light (365 to 405 nm) and that the material and resultant degradation by-products are biocompatible (*26*, *27*). The use of these compliant light-triggerable hydrogels has, however, been restricted to in vitro encapsulation and release of living cells, proteins, and model therapeutics for studies of cell mechanics in physiological and pathological states (*28*, *29*). In vivo use of light-degradable hydrogels has thus far been precluded by the low elasticity and strength of these materials. Moreover, in vitro studies of cell mechanics require laser-induced degradation of these gels, resulting in selective patterning of microscale regions with different geometric and material properties. As a result, there has been limited exploration of bulk degradation of these polymers from nonlaser light sources, which is necessary for their implementation in vivo. Developing a robust, biocompatible, and light-cleavable hydrogel with bulk-responsive functionality could enable the use of these materials in a range of in vivo applications that require dynamically triggerable degradation of an implantable device.

## RESULTS

### Synthesis scheme for light-triggerable hydrogels

**Fig. 1:**
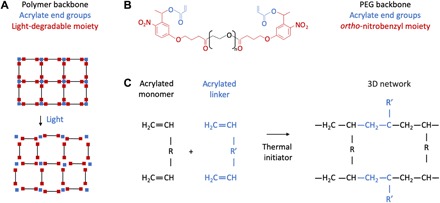
Light-degradable hydrogel synthesis scheme. (**A**) Schematic of light-degradable 3D hydrogel network. (**B**) Custom-synthesized acrylated oNB linker. (**C**) Schematic of linker integrating with any polymer network that relies on acrylate-based radical polymerization.

Acrylate functional groups at each end of a monomer chain readily connect to form cross-linked three-dimensional (3D) networks through radical polymerization (*30*). This method of forming hydrogel networks has formed the basis of a large body of literature on microfabrication and 3D printing (*31*, *32*). A polymer that contains a light-cleavable oNB moiety and is capped by acrylate functional groups can thus serve as an optically responsive linker for any polymeric network that relies on acrylate-based cross-linking (Fig. 1A). Light-triggered degradation of the linker results in on-demand dissolution of the network. Moreover, the oNB linker can be mixed in different proportions with acrylated linkers that are not responsive to light, rendering tunability of degradation from partial to complete dissolution of the 3D polymer network.

We custom-synthesized an oNB linker that matched these requirements using a commercial custom chemical synthesis manufacturer and a previously reported chemical structure, PEG 4000 4-(3-(1-acryloyloxyethyl)-4-nitrophenoxy) butanoate (Fig. 1B and fig. S1) (*27*). Previous reports of this chemical structure used it as a backbone to form purely PEG-based and extremely compliant hydrogels, suitable for in vitro cell culture. We propose instead using this structure as a linker to connect acrylated monomers of different compositions and chain lengths, with pore sizes and mechanical properties determined by the ratio of acrylated monomer to acrylated linker (Fig. 1C). A thermal initiation process, triggered by ammonium persulfate (APS) and *N*,*N*,*N*′,*N*′-tetramethylethylenediamine (TEMED), can be used to form cross-linked 3D networks that are light cleavable. This mechanism has been used extensively with acrylated linkers such as *N*,*N*′-methylenebisacrylamide (MBAA) but has not previously been reported with an optically responsive linker, such as the oNB linker we have synthesized. We hypothesize that a polymer synthesized in this way will provide both the robustness and triggerability required to use optical stimuli as dynamic triggers for implantable devices.

### Mechanical and biocompatibility characterization of light-triggerable hydrogels

**Fig. 2:**
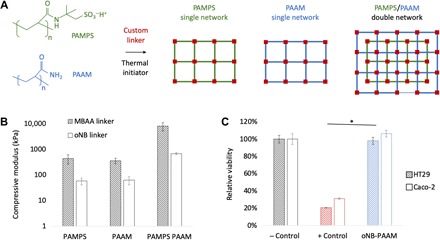
Mechanical and biocompatibility characterization of light-degradable hydrogels. (**A**) Light-degradable single- and double-network hydrogels can be synthesized from an array of biocompatible polymer backbones with acrylate functional groups, such as PAMPS and PAAM. (**B**) Mechanical properties of PAMPS, PAAM, and PAMPS/PAAM hydrogels with MBAA and oNB light-degradable linkers showing significant increases in mechanical properties in double-network hydrogels, as compared to single-network hydrogels (*n* = 3, *P* < 0.05). Results reported are for 1 M PAMPS with 4 mol % cross-linker (MBAA or oNB) and 2 M PAAM with 0.1 mol % cross-linker (MBAA or oNB). (**C**) In vitro toxicity study on two cell lines (HT29 and Caco-2) of PAAM network synthesized with light-degradable oNB linkers compared to negative control (− Control, untreated cells) and positive control (+ Control, cells treated with methanol). No significant negative effect of custom-synthesized light-degradable linker is observed (*n* = 4, *P* < 0.05).

The synthesis scheme we have developed is applicable for the formation of both single- and double-network gels. Double-network gels, which consist of interpenetrating networks of covalently bonded polymers, offer marked improvements in stretchability and toughness (*33*–*35*). Studies have demonstrated the enhanced mechanical properties of interpenetrating networks of poly(acrylamide) (PAAM) and poly(2-acrylamido-2-methylpropane sulfonic acid) (PAMPS) (Fig. 2A) (*4*). These PAMPS/PAAM networks are biocompatible and have been proposed as replacement materials for artificial cartilage in vivo (*10*). They are also transparent in the wavelength range of 300 to 800 nm (*36*), rendering them advantageous for use as a platform for engineering light-triggerable materials with bulk degradation properties.

The stiffness and stretchiness of PAMPS/PAAM gels can be regulated by varying the length and concentration of the monomer chains that form the backbone of the network, as well as the concentration of linker in the prepolymer solution (*37*). We have conducted optimization studies that tune these parameters separately and together (fig. S2A) and have shown that 1 M PAMPS with 4 mole percent (mol %) cross-linker (MBAA) with an interpenetrating network of 2 M PAAM with 0.1 mol % MBAA results in gels ~20 times stronger than single-network PAAM and PAMPS gels. Concentrations of APS (0.1 M) and TEMED (0.05 M) were also optimized, found to correspond with values previously reported in the literature (*27*), and kept constant throughout all hydrogel formulations reported below. We repeated these results with our custom-synthesized oNB linker replacing the MBAA linker, demonstrating a similar trend where the light-responsive double-network oNB-PAMPS/PAAM gel is ~12 times stronger than the single-network oNB-PAAM and oNB-PAMPS gels (Fig. 2B). Gels demonstrated similar trends in ultimate compressive strength, with MBAA-linked PAMPS/PAAM gels fracturing at 149 ± 49 kPa (strain at break 76 ± 4%) as compared to oNB-linked PAMPS/PAAM gels that fractured at 40.8 ± 3.2 kPa (strain at break 91 ± 1%). On average, hydrogels formed with the oNB linker are less stiff than those formed with MBAA, which we attribute to the increased chain length of the light-responsive linker. This chain length was mainly determined by the length of the PEG backbone of the linker, which corresponded to PEG of *M*_w_ (weight-average molecular weight) 4000, and was chosen to ensure miscibility of the linker in water. However, previous studies have shown that this miscibility can be retained with lower molecular weight PEGs, and reducing PEG molecular weight can result in increased stiffness of the polymer network (*9*, *38*–*40*). The compressive modulus of the oNB-linked gels we engineered was determined sufficient for our applications, as the maximum gastric pressure in humans ranges from 0.01 to 0.013 MPa (*41*, *42*), and the compressive strength of these hydrogels exceeds those values. However, the reduction of linker chain length offers an avenue for further improvements in mechanical properties if desired for other applications in future.

The cytotoxicity of our double-network gels was evaluated on two intestinal cell lines, HT29 and Caco-2, by incubating cells with gels before conducting a cell viability assay. The PAMPS/PAAM gels, with and without oNB linkers, were proven to be viable as compared to a positive control (Fig. 2C and fig. S2B).

### Tunable light-responsive behavior of triggerable hydrogels

**Fig. 3:**
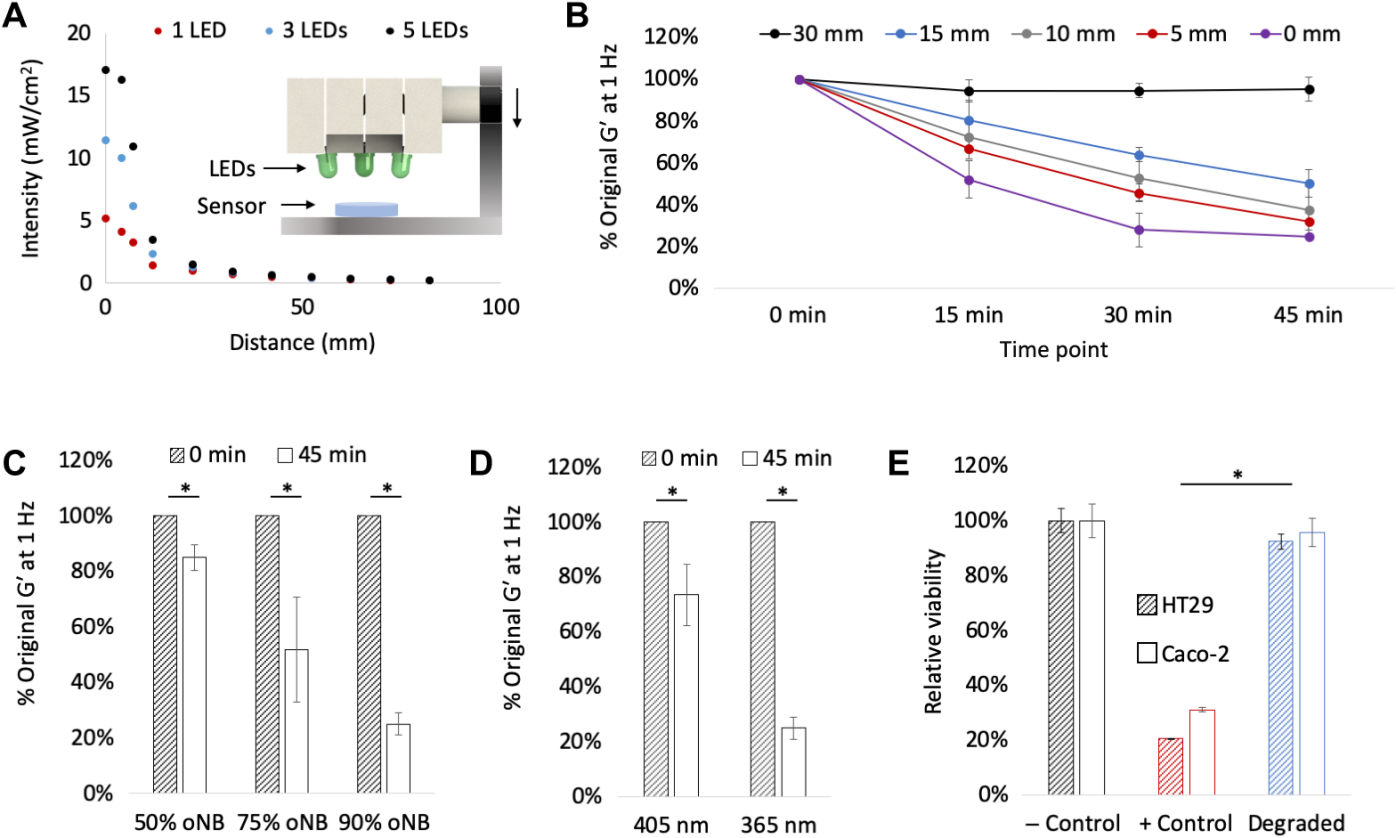
Tunable degradation of light-triggerable hydrogel. (**A**) Light intensity drops as a function of distance from the light source and the number of LEDs (365 nm). Inset: Schematic of array placement over light meter sensor. (**B**) Degradation of oNB-PAAM (4 M PAAM with 0.1 mol % oNB cross-linker) as a function of exposure time and distance from the three-LED array 365-nm light source (*n* = 3). (**C**) Effect of changing the percentage of oNB linker on ultraviolet (UV) light responsiveness of 4 M PAAM gels. Increasing the percentage of oNB linker leads to more significant drops in mechanical properties after 45-min degradation at 365 nm (*n* = 3, *P* < 0.05). (**D**) Effect of changing light wavelength from 365 to 405 nm. Shorter wavelengths lead to more significant drops in mechanical properties of 4 M PAAM gels with 0.1 mol % oNB cross-linker after 45-min degradation (*n* = 3, *P* < 0.05). (**E**) In vitro toxicity study on two cell lines (HT29 and Caco-2) of oNB-PAAM gels after degradation compared to negative control (− Control, untreated cells) and positive control (+ Control, cells treated with methanol), showing no significant effect of degradation by-products on cell viability (*n* = 4, *P* < 0.05).

The degree and timeline of light-triggered degradation can be tuned using a variety of parameters, including the intensity of the light source, the wavelength of the light source, and the oNB linker composition and distribution in the gel. To test the effect of each of these parameters on the mechanical properties of these gels, we constructed an in vitro platform for controlling the intensity of and distance from a light source. The platform enabled mounting between one and five light-emitting diodes (LEDs), emitting either 365- or 405-nm light, on a sliding rail (fig. S3A). A digital light meter was placed on the sample platen to measure the light intensity that would illuminate a hydrogel placed on the platen, and intensity was measured as a function of distance for sets of one, three, and five LEDs (365 nm) (Fig. 3A). Light intensity and distance should theoretically have an inverse square relationship, and this was corroborated by our results. Light intensity can also be precisely regulated by tuning the forward current applied to the LEDs (fig. S3B), with a maximum intensity of 17 mW cm^−2^ seen with a five-LED array operated at a forward current of 25 mA.

The dynamic degradation of oNB hydrogels in response to light illumination was monitored via rheological testing. oNB PAAM gels were irradiated with an array of three LEDs (365 nm) for 45 min, and the shear modulus, G′, was measured at 0, 15, 30, and 45 min (Fig. 3B). Light sources placed directly above the gels (0-mm distance) dropped in strength to 52, 28, and 25% of their original values after 15, 30, and 45 min of degradation, respectively. Increasing the distance from the light source to 5, 10, 15, and 30 mm resulted in decreases of the shear modulus to 32, 37, 50, and 95% of its original value after 45 min of degradation. This demonstrates a significant dependence of degree of degradation on the light intensity experienced by the gel, as the light intensity drops from nearly 11.4 mW cm^−2^ at a distance of 0 mm to 0.9 mW cm^−2^ at a distance of 30 mm.

The percentage of linker used in the oNB hydrogels determines the degree to which the gels can be degraded in response to light irradiation. Complete dissolution is attainable if only oNB linkers are used, but partial dissolution occurs when oNB linkers are mixed with other acrylated linkers. This reduction in mechanical strength, rather than complete degradation, could be useful depending on the target application of the material. Measurements of oNB-PAAM’s shear modulus at 0 and 45 min after irradiation were conducted on gels with 50, 75, and 90% oNB linker (Fig. 3C). In each case, MBAA served as a substitute for the remaining percentage of linker (50, 25, and 10%) required to form the 3D hydrogel network. As expected, increasing the percentage of light-cleavable linker in the hydrogel resulted in greater decreases in the mechanical strength of the gel following 45 min of 365-nm irradiation at 11.4 mW cm^−2^. The shear modulus of degraded gels dropped to 85, 52, and 25% of their original pre-degraded values for gels containing 50, 75, and 90% of oNB linker, respectively. Increasing the percentage of oNB linker in the hydrogels reduces the mechanical strength of the hydrogels in their pre-degraded state, as the MBAA linkers are shorter and stiffer than the oNB linker molecules (Fig. 2B). There is thus a trade-off between light responsiveness and peak mechanical strength that must be considered and tuned as applicable for each target application of this material. No significant changes in hydrogel swelling were observed in response to light-triggered degradation of gels containing 90% oNB linker (fig. S3C). A combination of surface erosion, which would result in thickness reduction, and bulk erosion, which would result in swelling increase, likely contributes to this phenomenon and is worth further investigation in future work.

Light wavelength is the final key parameter that regulates the triggerability of these materials. oNB linkers are responsive to blue light in the wavelength range of 365 to 405 nm and were predicted to be more readily cleavable when irradiated with higher-frequency optical stimuli. Both wavelength ranges have been proven to be cytocompatible by several studies investigating cells encapsulated in light-polymerized hydrogels (*31*, *32*, *43*). The shear modulus of gels following 45 min of irradiation at 11.4 mW cm^−2^ drops to 73 and 25% of its original value for gels irradiated with 405- and 365-nm light, respectively (Fig. 3D). On the basis of the sensitivity of a given in vivo system to light in the ultraviolet A (UVA) wavelength range and the time constraints for degradation in a target application, an appropriate wavelength can be chosen to suit the engineering design requirements.

The oNB linkers were proven to be biocompatible in a pre-degraded state (Fig. 2C), and cytotoxicity was reevaluated following light-triggered degradation to confirm that the chemical by-products of degradation were also cytocompatible (Fig. 3E). This served as validation that the oNB cross-linked gels are robust, triggerable, and safe to use for in vivo applications.

### Light-triggerable gels as dynamic triggers for GI devices

We identified GI devices as a compelling set of use cases for demonstrating the advantages of a dynamic light-activated trigger in a medical implant. These devices have a range of functional applications, ranging from prolonged drug delivery to in vivo sensing to space filling for bariatric applications (*15*, *17*, *44*). Many devices that accomplish GI residence in vivo have been developed and implemented, and their degradation over time is determined by the devices’ material properties before implantation (*45*, *46*). The ability to trigger the degradation of gastric-resident devices on demand could greatly enhance the tunability and safety of procedures that use these devices (*47*). Triggered dissolution through exposure to a safe ingestible trigger could transform our capacity to remove devices from the GI tract without the need for an endoscopic or surgical intervention. For example, bariatric balloons are currently used to fill space inside the stomach and modulate satiety, thereby limiting food intake. Currently, these devices are removed endoscopically. Stents are used throughout the GI tract, and temporary stents in the major segments of the GI tract are mechanically displaced and removed via an endoscopic procedure. Dynamically degrading these balloons in vivo without an invasive procedure would offer a substantial advantage over the state of the art but has not been demonstrated. Here, we present two examples of the application of our light-triggerable hydrogel system in the GI tract: a light-triggerable bariatric balloon and a light-triggerable stent.

### Light-triggerable gels as dynamic triggers for bariatric balloons

**Fig. 4:**
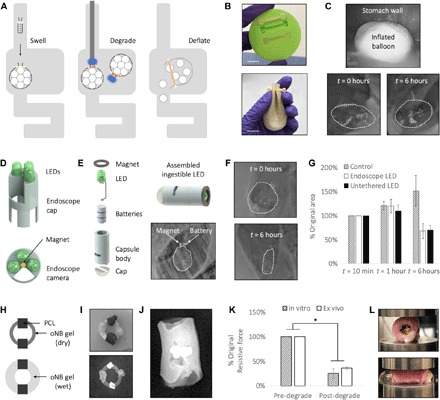
Light-degradable hydrogels as dynamic triggers in GI devices. (**A**) Schematic of balloon insertion and inflation (left), degradation via either an endoscopic or untethered LED light source (middle), and subsequent deflation (right). (**B**) Casting of oNB-PAAM gel pins (top) and assembled balloon sealed with a cast gel pin (bottom). Photo credit: Ritu Raman, MIT. (**C**) Balloon is inserted through the esophagus and swells in the stomach as observed endoscopically 1 min after insertion (top) and radiographically (bottom) immediately after insertion and after 6 hours in vivo. (**D**) Design of LED cap that can be attached to the inserted end of an endoscope. Wires to power the LEDs are threaded through the endoscope, and a hole in the array maintains visibility through the endoscope’s integrated camera. A magnet at the center of the array enables docking to the metallic piece attached to the sealed end of the balloon. (**E**) Design of ingestible pill-shaped LED. Computer-aided design (CAD) rendering shows assembly process for ingestible LED: Batteries, LED, and magnet are inserted into 3D-printed hollow cylindrical body and sealed with epoxy into a water-tight device. LED is turned on when a metal conductive tab is pushed into the slit in the side of the device. Magnetic docking of the LED to the balloon in vivo is observed radiographically (bottom right). (**F**) After the light-triggerable oNB-PAAM gel pin is degraded, the filler leaks out and the balloon decreases significantly in size as observed radiographically at *t* = 0 hours (top) and 6 hours (bottom). (**G**) The balloons degraded using both the endoscopic LED array and untethered LED decreased significantly in size at *t* = 6 hours as compared to a control (*n* = 3, *P* < 0.05), indicating successful on-demand activation of the oNB-PAAM gel trigger. (**H**) Schematic of esophageal stent device composed of an oNB-PAMPS gel ring with PCL beads. (**I**) Photograph (top) and radiographic image (bottom) of the assembled device. PCL beads are painted with a barium sulfate paint to increase visibility via x-ray. Photo credit: Ritu Raman, MIT. (**J**) The assembled device is placed inside an ex vivo esophagus, and swelling of the device ensures a press fit with the tissue that withstands compression. (**K**) Reduction in the esophageal stents’ resistive force to external compression in vitro and ex vivo after light-triggered degradation (*n* = 3, *P* < 0.05). (**L**) Top: Following degradation with the endoscopic LED array described in (D), the gel changes color from clear to orange, an indicator of degradation as observed in fig. S6. Bottom: The degraded gel leaks out of the esophagus when the tissue is compressed to half its original width commensurate with esophageal peristaltic movement in vivo. Photo credit: Ritu Raman, MIT.

Engineering a gastric-resident balloon involves several design challenges including the ability to function at body temperature in the highly acidic, humid, and bacteria- and enzyme-rich environment of the stomach. The device must be small enough to pass through the esophagus and accomplish a shape change in the stomach to prevent passage through the pylorus into the small intestines. To maintain gastric residence, it must withstand the peristaltic forces, on the order of 3 N, that aid digestion of food in the GI tract. A method of targeted light-based degradation of the gel must be accomplished in the gastric environment, and this must, in turn, trigger a shape change in the balloon that promotes its passage through the pylorus and into the intestines (Fig. 4A). We designed a space-filling balloon, composed of a stretchy porous polymer shell and a filler that rapidly inflates when wet. An oNB hydrogel cast in a capped pin shape weaves through the open end of the balloon, sealing it and preventing the inflating filler from leaving the balloon’s polymer shell (Fig. 4B and fig. S4). We chose a strong but stretchy composition for our light-triggerable hydrogel, composed of 4 M PAAM cross-linked with 0.1 mol % oNB, based on the mechanical requirements of this application.

In vitro tests of balloon swelling kinetics in simulated gastric fluid (SGF) enabled optimization of a variety of parameters including the degree of porosity of the polymer shell (fig. S5A) and the composition of the filler material (fig. S5, B and C). Final design parameters that optimized the rapidity of balloon expansion, the degree of balloon swelling, and the retention of the swollen shape over time were chosen. Latex membranes were modified to include an array of 300-μm pores and filled with 150 mg of sodium polyacrylate and 1350 mg of PolySnow. These materials have well-established biocompatibility profiles (*48*–*50*). The balloons swelled 22 times their original volume to 71 ml and retained their size up to 24 hours after immersion in SGF (fig. S5D). To test whether the inflated balloon could withstand the compressive peristaltic forces of the stomach, we conducted in vitro cyclic compression tests on the device up to 24 hours after swelling and observed no mechanical damage to the polymer shell or the oNB hydrogel during compression, as well as no filler leakage for compression forces up to 10 N (fig. S6A). A central point of the hydrogel pin was irradiated with 365-nm light at 11.4 mW cm^−2^ for 30 min (fig. S6B), and the balloon was tested again via cyclic compression (fig. S6C). Following activation of the light-activated trigger for 30 min, the weakened degraded hydrogel failed to fully seal the balloon. The filler leaked out of the balloon at compressive forces of 3 N, commensurate with the degree of peristalsis in the gastric environment. This indicated that the devices we had designed could accomplish a shape change in the stomach to promote gastric residence, maintain that shape change and mechanical integrity in the acidic stomach environment, and be readily degraded on-demand with a light stimulus to drive passage through the pylorus into the intestines and out of the body.

In vivo tests of this device were conducted in Yorkshire pigs (65 to 85 kg in weight), as their GI tract anatomical dimensions are similar to those of human adults (*51*). Assembled and sealed balloons were inserted through the esophagus into the stomach. Immediate and retained swelling was confirmed via endoscopic and radiographic imaging (Fig. 4C and movie S1). Radiographic imaging was enabled by dispersing small metal beads throughout the filler to ensure radiographic visibility of the balloon on an x-ray. Residence without a significant reduction in size was confirmed before in vivo tests of light-triggered degradation of the device. An endoscope was modified to include an end cap that included three LEDs (365 nm), producing a maximum light intensity of 11.4 mW cm^−2^ (Fig. 4D and fig. S7A). A small magnet was added to the center of the cap, enabling coupling with a small metal piece attached to the oNB pin-sealed end of the balloon. The endoscope was navigated toward the balloon, enabling magnetic docking (movie S2). Once docking was confirmed, the LED array was turned on and left to irradiate the central portion of the oNB gel pin for 30 min. To prove that a dynamic light trigger could be activated via an ingestible light source, rather than an invasive endoscopic procedure, we also designed a battery-powered ingestible LED capable of magnetic docking with our balloon devices (Fig. 4E and fig. S7B). The materials and methods used to fabricate the ingestible LED have well established biocompatibility profiles (*52*–*54*). The LEDs demonstrated successful docking with the balloons (Fig. 4E and movie S3) and were left on for at least 70 min, rather than 30 min, to account for the reduction in light intensity from 11.4 to 5.19 mW cm^−2^ while keeping the energy density constant (25.2 J cm^−2^). LED docking occurred within minutes of being placed inside the stomach, as both the balloon and the LED floated in gastric fluid and stomach peristalsis brought the devices into a degree of proximity that activated magnetic attraction of the LED to the balloon. After light-triggered degradation through either mechanism, the balloon was left in the stomach and imaged again after 6 hours, showing a significant reduction in size (Fig. 4F). For both the endoscopic and ingestible light sources, alternate designs that enable illumination from further distances or balloon docking enabled by mechanical interlocking can be readily manufactured and implemented, if required. Note that if such devices were to be used in humans, then further studies would need to be conducted to determine the average time to docking, which will likely depend on a variety of parameters including the size of the stomach, which varies between individuals and between fed and fasted states, as well as the contents of the stomach.

Balloons from the nondegraded control, endoscopic LED-degraded, and untethered LED-degraded groups were imaged radiographically after initial insertion and swelling, 1 hour after insertion (immediately after degradation of the oNB gel pin), and 6 hours after insertion. Control balloons increased in size to 150% of their original volume, indicating that swelling kinetics of the balloon in vivo are slower than observed in vitro, resulting in a slower increase to the maximum volume of the device. Considering that devices in vivo are not fully immersed in gastric fluid, while our in vitro tests involved complete submersion of the balloons in SGF, this result can be readily explained. Balloons whose oNB gel pin seals were activated by both the endoscopic LED and untethered LED groups decreased in size significantly after degradation to 68 and 70% of their original values immediately after insertion (Fig. 4G). This indicates that on-demand degradation of the device can be triggered by a noninvasive light stimulus and that gastric peristaltic forces are sufficient to drive device filler leakage. This is the first demonstration of the use of light-degradable hydrogels in vivo, to our knowledge. Moreover, this serves as a compelling demonstration that an ingestible trigger can drive on-demand passage of a gastric-resident device, reducing the need for invasive endoscopic procedures.

### Light-triggerable gels as dynamic triggers for an esophageal stent

Light-triggerable hydrogels are potentially applicable in a variety of in vivo applications beyond gastric-resident devices. We manufactured a simple esophageal stent, designed for deployment in the esophagus for potential structural support and/or local drug delivery. To target this application, we cast a simple cylindrical ring of oNB hydrogel with beads of ﻿poly(e-caprolactone) (PCL), a polymer that has been used extensively for in vivo controlled drug release (Fig. 4H) (*15*, *55*). We chose a stiff hydrogel with a large swelling ratio for this use case, composed of 4 M PAMPS cross-linked with 4 mol % oNB linker.

Light-triggerable hydrogels of this composition were cast in half-ring molds, dried, and looped through PCL beads before being sealed into a full ring shape (Fig. 4I and fig. S8A). The beads were painted with a barium sulfate mixture to make them radiopaque, rendering them visible via x-ray inside an ex vivo swine esophagus (Fig. 4J). Preliminary in vitro mechanical tests of the oNB hydrogel stent were conducted to determine their resistance to compression to 50% of their original diameter, commensurate with the deformation they would experience in response to esophageal peristaltic forces in vivo (fig. S8B). Degradation was accomplished via light activation of the oNB trigger for 30 min using an esophageal LED array (Fig. 4D). In vitro tests revealed that the devices’ resistance to compression dropped to 25% of its original value after degradation (Fig. 4K). Similar tests were conducted ex vivo by compressing an esophagus with a stent placed inside, revealing that light-triggered reduction in mechanical strength of the gel scaffold resulted in a drop in resistive force to 36% of the pre-degraded value. Values for in vitro and ex vivo tests were not found to be significantly different after degradation. Moreover, ex vivo tests revealed that compression after degradation led to complete collapse of the stent and ejection out of the esophagus (Fig. 4L). This indicates that if these devices are used in vivo in the future, then they would remain lodged in the esophagus until degraded, after which time peristaltic waves through the tissue tube would force the device into the stomach, to be passed through the GI tract. An endoscopic procedure used to monitor the therapeutic effectiveness of the esophageal device, by examining the reduction in the size and severity of throat ulcers, for example, could also be used to safely irradiate and noninvasively remove the device from its in vivo setting without risking laceration of the newly formed tissue.

## DISCUSSION

There is a critical need for a safe, noncontact, and dynamically activated trigger for actuating implantable devices. Inducing on-demand degradation of these devices enables tuning treatments to the needs of individual patients, diseases, and recovery schedules. Triggers based on other stimuli, such as heat, pH, and hydrolytic or enzymatic chemicals, have been used for this purpose in some in vivo applications but come with many restrictions that limit their use for several disease use cases. Chemical stimuli require contact with the triggerable material, pH stimuli can only be used in certain bodily environments and can be affected by commonly used therapeutic drugs such as proton pump inhibitors, and heat-based stimuli can have off-target effects on surrounding tissue. Light-triggerable materials offer noncontact on-demand degradation with high spatial and temporal resolution (*56*). We have developed a tough light-triggerable hydrogel material with tunable mechanical properties and modular design that can be degraded in a safe and noncontact manner in a variety of bodily locations. This is, to our knowledge, the first demonstration of light-degradable hydrogels in vivo.

To develop a light-triggerable material with the mechanical robustness and versatility required for in vivo use, we custom-manufactured an acrylated oNB-based compound and used it as a linker to polymerize 3D hydrogel networks from a range of materials, including PAAM and PAMPS single-network gels and tough PAMPS/PAAM double-network gels. We showed that the mechanical properties and degradation timelines of these light-triggerable materials could be tuned using a variety of parameters, such as gel composition, light wavelength, intensity, and distance from the material. The mechanical properties of our material far exceeded previously reported properties for light-triggerable gels and enabled their in vivo use. The material and its degradation by-products were proven to be biocompatible, and use of wavelengths in the 365- to 405-nm range has been well established in previous literature as safe for use with cells (*31*, *32*, *43*). If future in vivo applications involve cells that are more sensitive to shorter wavelength (365-nm) optical stimuli, 405-nm light can be used as the wavelength of choice with a corresponding increase in exposure time.

We showcased the advantageous properties of our light-triggerable material in two model use cases, a gastric-resident bariatric balloon and an esophageal stent. In the case of the balloon, we were able to trigger a light-activated reduction in the size of an ingestible space-filling balloon in vivo. If used in a bariatric patient, this functionality could enable on-demand passage of the space-filling device when gastric residence is no longer required. This would provide a significant advantage over the current clinical standard, which requires an invasive endoscopic procedure to retrieve the balloon from the stomach, rather than the facile ingestion of a light-emitting pill. Because heat- or chemical-based stimuli would require complete submersion of the balloon in a triggering liquid, and pH-based stimuli would require offsetting the carefully regulated pH of the gastric environment, light-based stimuli offer a significant advantage in dynamic tuning degradation timelines of this type of device. This has potential for significant clinical impact as, in North America alone, more than two of three adults are overweight or obese, and projections estimate that over 85% of adults fall into these categories by 2030 (*57*). Similarly, for the esophageal stent device, we have developed that the light-based trigger provides a noncontact method for safely removing the stent from the lower esophageal sphincter without risking off-target detrimental effects to the esophageal lining that could arise from mechanical-, heat-, or chemical-based triggers. This could be used to treat benign and malignant stenoses and strictures.

These devices target just two of a range of potential uses for the light-triggerable hydrogel we have developed in critical global public health challenges. For both cases, we tuned the composition of the material to match the mechanical requirements of the target applications, showcasing the modularity and versatility of our platform. Likewise, the oNB hydrogel can be readily cast in a variety of shapes, enabling simple adaptation and prototyping of device design. The material properties, device shape, degradation timeline, and method of delivering light stimuli can all be individually tuned to suit different clinical needs. Previous studies of photodynamic therapy in the body using light-emitting thin films or optical fibers can, for example, be used in conjunction with our material to develop light-responsive implantable devices for a range of applications (*58*, *59*). Moreover, optical stimuli can be combined with other types of stimuli to enable materials capable of responding to a range of environmental signals, as required (*19*, *60*). By implementing light-degradable hydrogels in vivo and demonstrating their degradation on-demand, we provide biomedical engineers and clinicians with a previously unavailable, preclinically and preliminarily safe, and precise tool to design dynamically actuated implantable devices.

## METHODS

### Formulation and casting of light-triggerable single- and double-network hydrogels

A light-sensitive oNB moiety was converted into a chemical linker through functionalization with acrylate groups at each end of the moiety. The molecule, PEG 4000 4-(3-(1-acryloyloxyethyl)-4-nitrophenoxy) butanoate, was custom-synthesized by Lianyungang Tengfa Bio-Tech Co. Ltd. It was stored in the dark at 4°C when not in active use. Single- and double-network hydrogels were formulated using two types of acrylated monomers: 2-acrylamido-2-methyl-1-propanesulfonic acid (AMPS; Sigma-Aldrich) and acrylamide (AAM; Sigma-Aldrich). Nontriggerable formulations of the gels were polymerized using the cross-linker MBAA (Sigma-Aldrich), and the polymerization process was triggered by the thermo-initiator APS (Sigma-Aldrich) and the accelerator TEMED (Sigma-Aldrich). The concentration of the monomers, AMPS and AAM, was varied between 1 and 4 M, and the concentration of MBAA was varied between 0.1 and 4 mol % with respect to the monomer. The concentrations of APS and TEMED were optimized to 0.1 and 0.05 M, respectively, and then kept constant across hydrogel formulations. Double-network gels were prepared by first polymerizing a PAMPS hydrogel, followed by immersing the gel in a PAAM solution for 1 day before triggering polymerization, enabling the formation of an interpenetrating network. Light-triggerable gels were formulated by replacing all or part of the MBAA linker with the custom-synthesized oNB linker. Gels used for mechanical testing and in vitro characterization were immersed in water for several hours before testing to ensure complete swelling of the networks. Gels used for assembling in vivo and ex vivo devices were air-dried before assembly and accomplished swelling in the context of their target use application.

### Biocompatibility testing of light-triggerable hydrogels

Hydrogels were prepared and incubated in well plates seeded with either HT29 or Caco-2 cells (American Type Culture Collection). Cells were maintained in culture in Dulbecco’s modified Eagle’s medium (Life Technologies) supplemented with 10% fetal bovine serum (Life Technologies), 1% nonessential amino acids (Life Technologies), and 1% penicillin-streptomycin solution (Life Technologies). Following incubation of the gels in the cell-containing wells, 100 μl of medium was extracted from the wells, mixed with 10 μl of alamarBlue reagent (Life Technologies), and incubated at 37°C for 1 hour. Fluorescence (560-nm excitation, 590-nm emission) of this fluid mixture was measured using a microplate reader (﻿Infinite M200Pro, Tecan). The readings were compared to a positive control, prepared by treating cells with methanol for 1 hour before the cytotoxicity assay, and a negative control, prepared from untreated cells.

### In vitro degradation and mechanical characterization of light-triggerable hydrogels

Mechanical characterization of gels was conducted on cylindrical gels (8 mm diameter, 3 mm depth) cast in custom-made molds. The molds were cast using a flexible silicone (Elite, Zhermack) around a 3D-printed template manufactured with a Formlabs Form 2 machine. The compressive modulus of the hydrogels was measured on an Instron 5943 mechanical testing apparatus. Hydrated gels were placed on a flat loading platen and compressed at a strain rate of 0.1 mm s^−1^ until they fractured. The shear modulus of hydrogels was measured on a TA Instruments AR2000 rheometer. Strain sweeps between 0 and 10% were conducted at a constant frequency of 6.283 rad s^−1^ to determine the linear regime of the material. Frequency sweeps were conducted between 0.1 and 100 rad s^−1^ at a constant strain of 2.86%. Values of G′ at 0.1 rad s^−1^ were compared from frequency sweeps across samples to compare mechanical performance between different gels. In vitro degradation of the gels was conducted using a custom-built apparatus consisting of a LED housing fixture, 3D-printed using Stratasys Objet30 Pro with VeroClear material, and attached to a sliding rail. The fixture could hold between one and five LEDs, and its distance from the irradiated sample could be tuned with millimeter precision. LEDs of two wavelengths, 365 nm (Marktech) and 405 nm (Bivar), were used in these studies. Samples were placed between 0 and 30 mm away from the light source, and the light intensity of the LEDs was measured using a light meter (Sper Scientific Direct). Samples were stored and degraded in a custom-modified room in which all other light sources were modified to remove wavelengths in the blue and UV range.

### Manufacture and in vitro testing of light-triggerable gastric-resident balloons

The polymer shell of the gastric-resident devices was formed from latex balloons (30 cm diameter; Amazon). The balloons were stretched around a 100-ml round bottom flask (Sigma-Aldrich) and mounted on a rotary tool in a laser cutting machine (Universal Laser System). A circumferential pattern of holes (250 to 400 μm) spaced 1.25 mm apart was created in Adobe Illustrator and sent to the laser cutter’s processing software, Universal Control Panel. The laser settings were tuned to 40% power, 40% speed, and 500 dots per inch (DPI). Following calibration of the balloon location, the exhaust pump was turned on for ventilation and the engraving operation was executed. The balloon was removed from the flask and filled with 1500 mg of a rapidly inflating mixture, composed of sodium polyacrylate and PolySnow (Flinn Scientific) in varying percentage compositions between 50 and 100% PolySnow. The devices were sealed by threading through the open end of the balloon with a dried cast gel pin (4 M PAAM, 0.1 mol % oNB linker). The pin (2.5 mm width, 2.5 mm depth) was prevented from sliding off the balloon by capped ends (6 mm width). The pins were fabricated from custom-made silicone molds cast from a 3D-printed template (Stratasys Objet30), as described above, and air-dried for 1 day before device assembly. Following device assembly and before in vitro mechanical testing, the balloons were swelled in SGF (pH 1.2) for at least 1 hour. In vitro mechanical testing of the balloons was conducted on Instron 5943 at a strain rate of 2 mm s^−1^ at compression forces between 3 and 10 N.

### Developing instrumentation for in vivo activation of light trigger

The housing for our endoscopic LED array was designed in computer-aided design (CAD) software (SOLIDWORKS) and 3D-printed on a Formlabs Form 2 printer using Formlabs Durable resin. The housing included three slots for LED leads, a slot for a cylindrical magnet, and a viewport for the endoscope’s camera. The three LEDs (365 nm; Marktech) were placed through the slots and wired in series by twisting the leads of neighboring LEDs together. Wires long enough to be strung through the working channel of the endoscope were then soldered to the positive and negative end leads. The cavity containing all the electrical connections was filled with MED3-4213 silicone adhesive (NuSil) to ensure that the device would be protected from fluid in vivo. Any gaps between the LEDs and the housing were also sealed with the same silicone adhesive.

The capsule body for the ingestible LED was designed using CAD software (SOLIDWORKS) and printed in two parts on a Formlabs Form 2 printer using Formlabs Durable resin. The leads of the LED (365 nm; Marktech) were bent and trimmed to fit around three size 10 hearing aid batteries (Energizer). The LED’s negative lead was placed in the bottom half of the capsule, followed by one of the three batteries. A copper tab wrapped in tape was then placed through the slot on the side of the housing to cover the battery and disrupt the electrical connection, ensuring that the LED would not turn on during fabrication. The remaining batteries were loaded into the bottom half of the housing, and the rest of the LED was bent into place so that everything was arranged in an inline configuration. The top half of the housing was press-fit into the bottom half, encapsulating the electronics. Two stacked ring magnets (SM Magnetics) were glued onto the top of the capsule, surrounding the LED, and the gap between the LED and the magnets was filled with MED3-4213 silicone adhesive to protect the electronics from fluid in vivo.

### In vivo testing of light-triggerable gastric-resident balloons

Animal experiments were approved by the Committee on Animal Care at the Massachusetts Institute of Technology and conducted on Yorkshire pigs (65 to 85 kg). This model has been used extensively in the literature, as the GI anatomical dimensions of these animals are similar to those observed in humans. Pigs were sedated [using Telazol (5 mg/kg, intramuscularly), xylazine (2 mg/kg, intramuscularly), and atropine (0.04 mg/kg, intramuscularly)], intubated, and maintained with isoflurane (1 to 3% in oxygen) before insertion of an overtube placed under the visual guidance of an endoscope (US Endoscopy), as previously described (*15*–*17*). Assembled balloon devices were inserted through the overtube, and videos of inflation in gastric fluid were procured using endoscopic videography. The overtube was removed once the devices were administered. The filler for these balloons was modified from those used for in vitro testing by mixing 1.2-mm-diameter metal beads (McMaster-Carr) into the filler to enable radiographic imaging. A small metal tab was incorporated near the region of the hydrogel requiring light illumination to enable magnetic docking of the endoscopic and ingestible LED to the sealed end of the balloon. The endoscopic LED array was navigated through the overtube and, once magnetic docking with the balloon was established, turned on for 30 min, emitting a light intensity of 11.4 mW cm^−2^. The ingestible LED array was held with biopsy forceps and navigated through the overtube toward the balloon to enable rapid magnetic docking with the balloon in vivo. It was turned on before insertion into the animal and emitted a light intensity of 5.19 mW cm^−2^. Implanted balloons were endoscopically and radiographically imaged immediately following insertion and inflation, immediately after degradation (~1 hour after insertion), and at the end of the experiment (~6 hours after insertion). Their relative sizes were measured using ImageJ software to study the effects of light irradiation on the triggerable devices.

### Manufacture and ex vivo testing of light-triggerable esophageal stent

The two halves of the rings that composed our annulus-shaped esophageal stent (10 mm diameter, 1 mm thickness) were formed by injecting hydrogels (4 M PAMPS, 4 mol % oNB) into molds, prepared as described in previous sections using a Formlabs 3D printer and Elite flexible silicone (Zhermack). The ring halves were then air-dried for 24 hours before assembly with polymer beads formed from ﻿PCL (Sigma-Aldrich) cast cylinders with central holes created using a drill press. The beads were painted with barium sulfate to enable radiographic imaging once inserted into esophageal tissue. The devices were sealed into a completed ring using the same hydrogel formulation used to manufacture the two halves of the stent device. Assembled devices were immersed in phosphate-buffered saline (PBS) for 1 hour before in vitro mechanical testing on Instron 5943. Devices were held by the PCL beads in grips and compressed to 50% of their undeformed diameter at a strain rate of 0.5 mm s^−1^. They were then degraded for 30 min using the endoscopic LED array described above and retested using the same parameters. Assembled devices were also inserted into ex vivo porcine esophagus tissue, followed by immersion in PBS for 1 hour, and imaged radiographically. Mechanical testing was carried out with the same degree of compression and strain rate, and the endoscopic LED array was inserted into the tissue’s central cavity to illuminate and degrade the stent over a period of 30 min. The devices’ resistance to compression, both in vitro and ex vivo, was measured by the load cell and compared across devices to determine the effect of light-triggered degradation.

## Supplementary Material

http://advances.sciencemag.org/cgi/content/full/6/3/eaay0065/DC1

Download PDF

Movie S1

Movie S2

Movie S3

Light-degradable hydrogels as dynamic triggers for gastrointestinal applications

## SUPPLEMENTARY MATERIALS

Supplementary material for this article is available at http://advances.sciencemag.org/cgi/content/full/6/3/eaay0065/DC1 Fig. S1. Schematic and characterization of light-triggerable linker. Fig. S2. Mechanical and biocompatibility characterization of tough hydrogel platform. Fig. S3. Characterization of light-induced hydrogel degradation in vitro. Fig. S4. Synthesis of gastric-resident balloon sealed with light-degradable hydrogel. Fig. S5. In vitro characterization of gastric-resident balloon swelling. Fig. S6. In vitro mechanical characterization of gastric-resident balloon before and after degradation. Fig. S7. Custom-manufactured light-emitting devices for in vivo triggering of light-degradable hydrogels. Fig. S8. Synthesis and characterization of light-triggerable esophageal stent. Movie S1. Balloon swelling in gastric environment in vivo. Movie S2. Endoscopic LED cap turning on in vivo. Movie S3. Demonstration of ingestible LED tethering to balloon in vivo.

## Appendix

View/request a protocol for this paper from *Bio-protocol*.
