# Supplementary material for: Light-degradable hydrogels as dynamic triggers for gastrointestinal applications
Source: Sci Adv. 2020 Jan 17;6(3):eaay0065. doi: 10.1126/sciadv.aay0065 (PMC6968934; doi:10.1126/sciadv.aay0065)
Supplement: http://advances.sciencemag.org/cgi/content/full/6/3/eaay0065/DC1 [file supp_6_3_eaay0065__index.html]

Science Advances | Science AdvancesAAASSearchScience AdvancesMenu

## Supplementary Materials

**The PDFset includes:**

- Fig. S1. Schematic and characterization of light-triggerable linker.
- Fig. S2. Mechanical and biocompatibility characterization of tough hydrogel platform.
- Fig. S3. Characterization of light-induced hydrogel degradation in vitro.
- Fig. S4. Synthesis of gastric-resident balloon sealed with light-degradable hydrogel.
- Fig. S5. In vitro characterization of gastric-resident balloon swelling.
- Fig. S6. In vitro mechanical characterization of gastric-resident balloon before and after degradation.
- Fig. S7. Custom-manufactured light-emitting devices for in vivo triggering of light-degradable hydrogels.
- Fig. S8. Synthesis and characterization of light-triggerable esophageal stent.

Download PDF

**Other Supplementary Material for this manuscript includes the following:**

- Movie S1 (.mov format). Balloon swelling in gastric environment in vivo.
- Movie S2 (.avi format). Ferrofluid filled into a long S-RuM architecture.
- Movie S3 (.avi format). Demonstration of ingestible LED tethering to balloon in vivo.

**Files in this Data Supplement:**

- Adobe PDF - aay0065\_SM.pdf
