# Supplementary material for: Light-degradable hydrogels as dynamic triggers for gastrointestinal applications
Source: Sci Adv. 2020 Jan 17;6(3):eaay0065. doi: 10.1126/sciadv.aay0065 (PMC6968934; doi:10.1126/sciadv.aay0065)
Supplement: Download PDF [file aay0065_SM.pdf]

Science Advances
AAAS

advances.sciencemag.org/cgi/content/full/6/3/eaay0065/DC1

# Supplementary Materials for

## Light-degradable hydrogels as dynamic triggers for gastrointestinal applications

Ritu Raman, Tiffany Hua, Declan Gwynne, Joy Collins, Siddartha Tamang, Jianlin Zhou, Tina Esfandiary, Vance Soares, Simo Pajovic, Alison Hayward, Robert Langer, Giovanni Traverso*

*Corresponding author. Email: cgt20@mit.edu, ctraverso@bwh.harvard.edu

Published 17 January 2020, *Sci. Adv.* **6**, eaay0065 (2020)
DOI: 10.1126/sciadv.aay0065

**The PDF file includes:**

Fig. S1. Schematic and characterization of light-triggerable linker.
Fig. S2. Mechanical and biocompatibility characterization of tough hydrogel platform.
Fig. S3. Characterization of light-induced hydrogel degradation in vitro.
Fig. S4. Synthesis of gastric-resident balloon sealed with light-degradable hydrogel.
Fig. S5. In vitro characterization of gastric-resident balloon swelling.
Fig. S6. In vitro mechanical characterization of gastric-resident balloon before and after degradation.
Fig. S7. Custom-manufactured light-emitting devices for in vivo triggering of light-degradable hydrogels.
Fig. S8. Synthesis and characterization of light-triggerable esophageal stent.

**Other Supplementary Material for this manuscript includes the following:**

(available at advances.sciencemag.org/cgi/content/full/6/3/eaay0065/DC1)

Movie S1 (.mov format). Balloon swelling in gastric environment in vivo.
Movie S2 (.avi format). Endoscopic LED cap turning on in vivo.
Movie S3 (.avi format). Demonstration of ingestible LED tethering to balloon in vivo.

*Supplemental Figures*

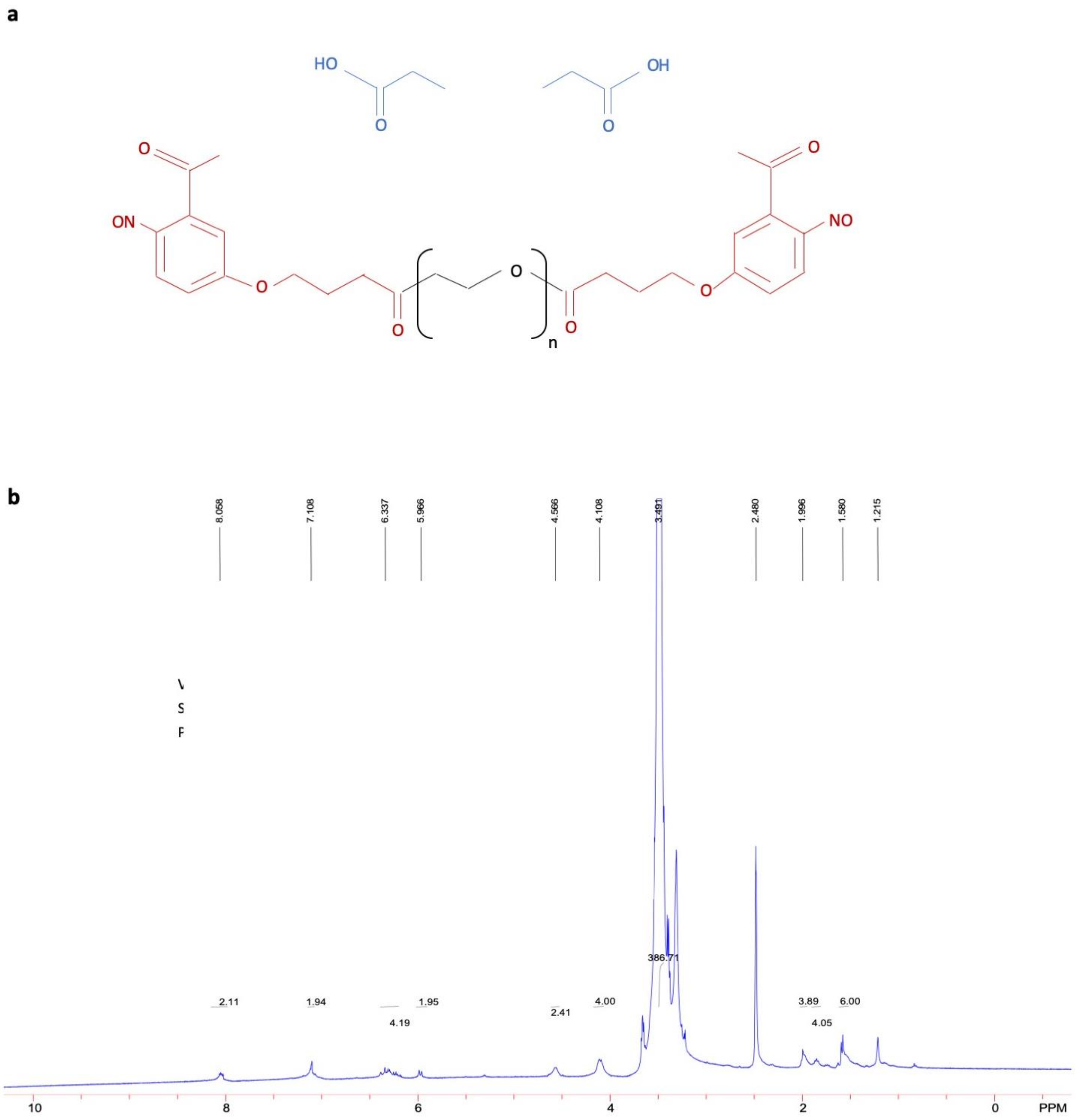


**Fig. S1. Schematic and characterization of light-triggerable linker.** (**a**) Chemical structure of degraded linker. (**b**) H NMR spectrum of custom-synthesized linker in DMSO solvent.

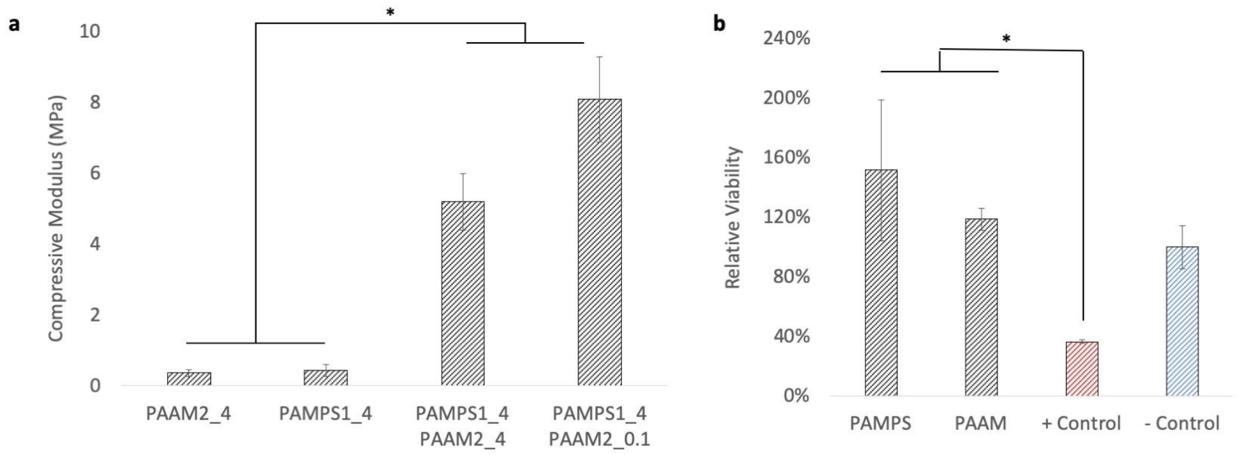


**Fig. S2. Mechanical and biocompatibility characterization of tough hydrogel platform.** (**a**) Optimization of composition of double network PAMPS/PAAM gels shows that compressive modulus can be significantly enhanced when the interpenetrating PAMPS network has a longer chain length, making it stretchier and increasing the strain at break ($n = 4$, $p < 0.05$). (**b**) *in vitro* toxicity study on HT29 cell line of PAAM and PAMPS networks with MBAA linkers. No significant negative effect of gels is observed ($n = 4$, $p < 0.05$).

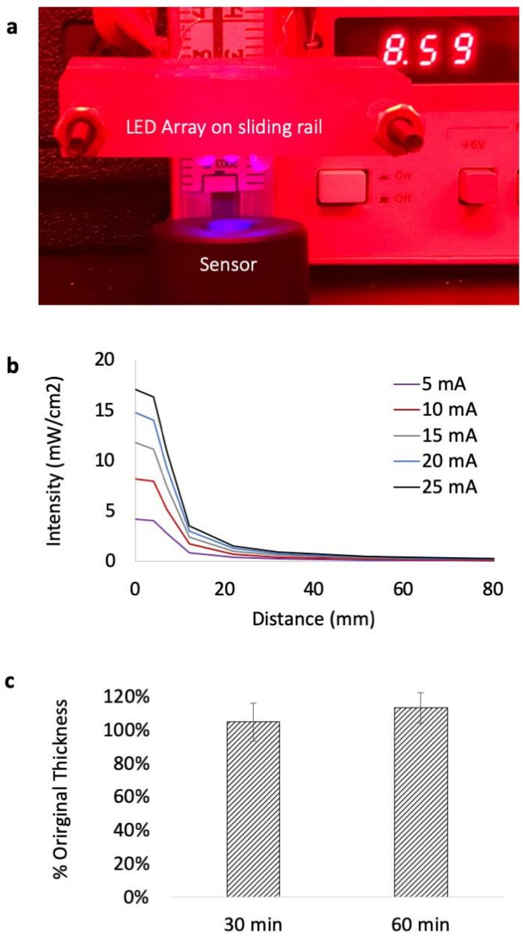


**Fig. S3. Characterization of light-induced hydrogel degradation in vitro.** (**a**) Schematic and picture of *in vitro* LED array with adjustable number of LEDs and distance from sample. Photo Credit: Ritu Raman, MIT. (**b**) Effect of increasing forward current on light intensity for 5 LED array, showing maximum power at the maximum forward current of the LED, as expected. **c)** Sample thickness pre- and post-degradation with 365 nm light at 11.4 mW $cm^{-2}$.

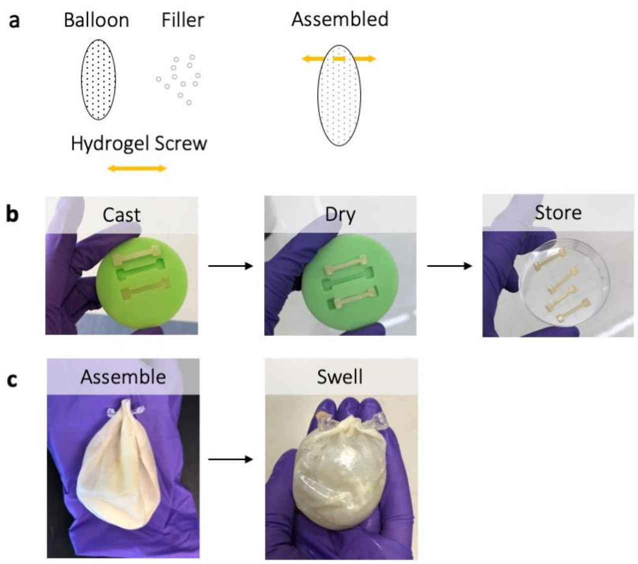


**Fig. S4. Synthesis of gastric-resident balloon sealed with light-degradable hydrogel.** (**a**) Schematic of proposed design and composition of gastric-resident balloon. (**b**) Fabrication process for injection molding capped oNB-PAAM gel pins that seal gastric resident balloon and prevent filler leakage. Photo Credit: Ritu Raman, MIT. (**c**) Assembly of balloons with filler inside and oNB gel pin seal (left) followed by immersion in SGF shows balloon swelling and filler retention (right). Photo Credit: Ritu Raman, MIT.

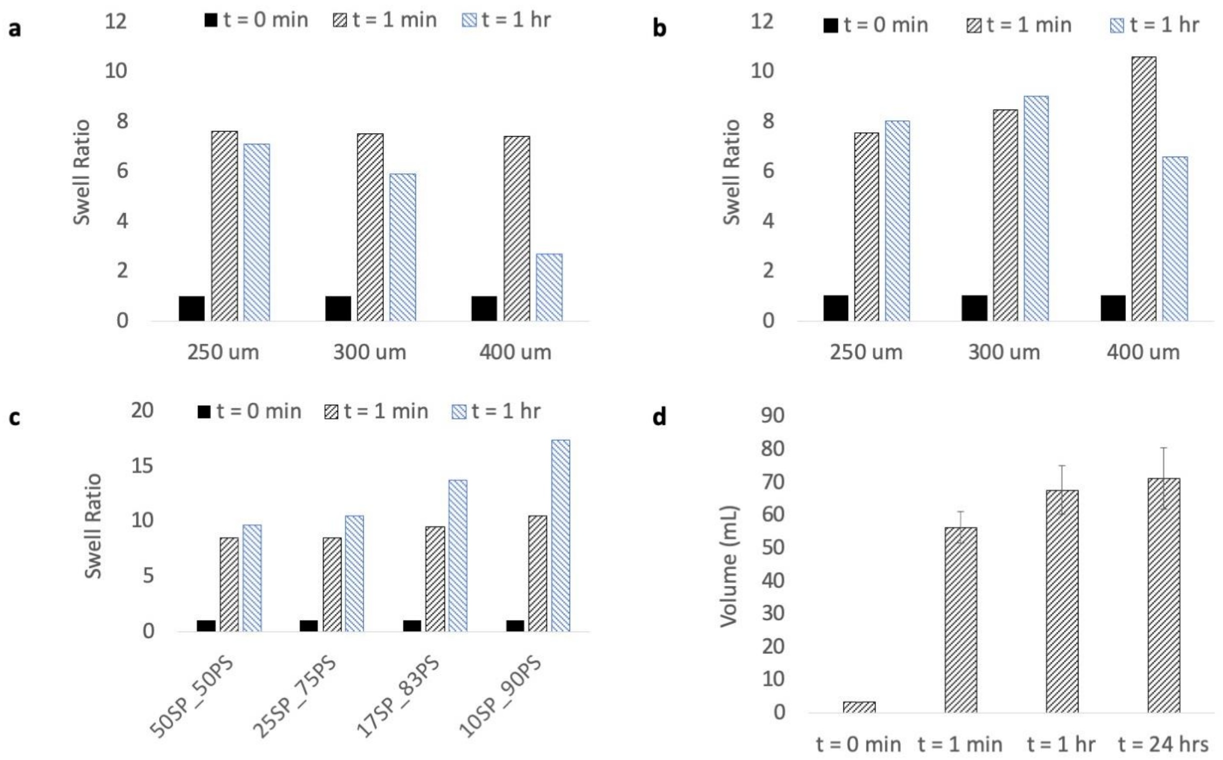


**Fig. S5. In vitro characterization of gastric-resident balloon swelling.** (**a**) Swelling ratio over time of balloons filled with 1500 mg PolySnow. Varying the diameter of the holes laser cut into the side of the balloons from 250-400 um shows that PolySnow leaks more quickly out of larger holes and the swelling cannot be retained over time. (**b**) Swelling ratio over times of balloons filled with 1500 mg filler composed of 25% sodium polyacrylate and 75% PolySnow. Swelling is retained in balloons with 250 and 300 um holes but not 400 um holes. (**c**) Optimization of filler composition by tuning sodium polyacrylate (SP) composition to 10, 17, 25, and 50% of the total 1500 mg filler volume. Greatest degree of swelling and retention is observed in filler composed of 10% SP and 90% PolySnow (PS). (**d**) Tracking volume of optimized balloon over time (300 um holes, 1500 mg filler with 10% SP and 90% PS) shows swelling up to ~22 times original volume over a period of one day.

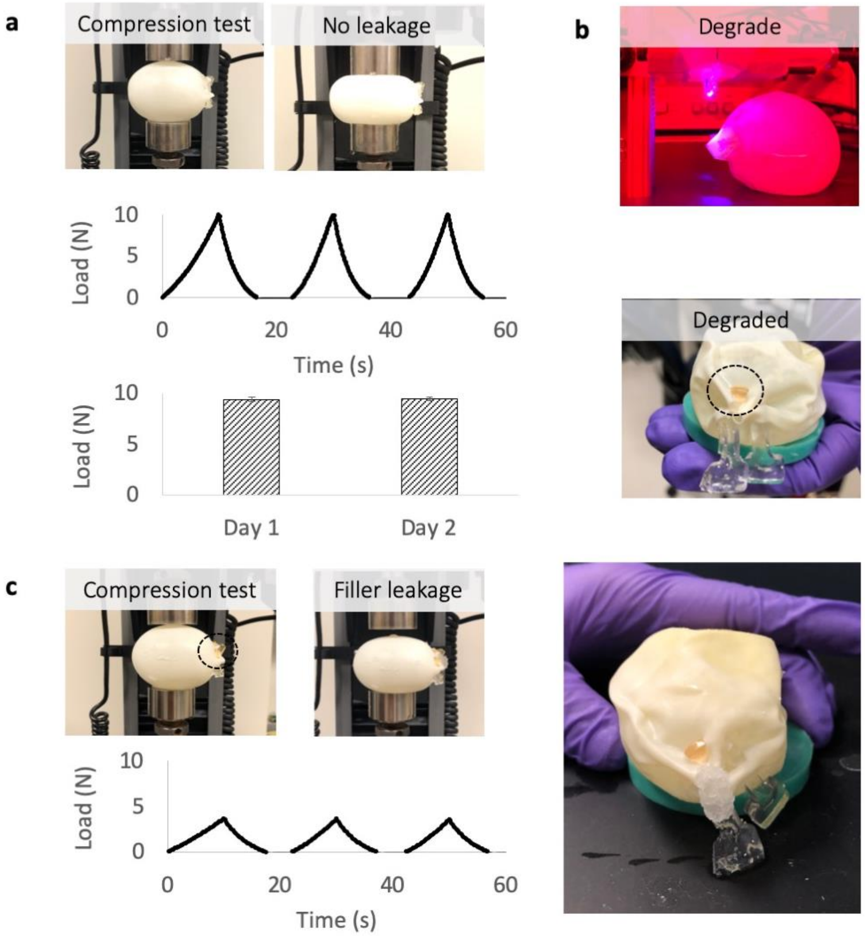


**Fig. S6. In vitro mechanical characterization of gastric-resident balloon before and after degradation.** (**a**) Compression test of inflated gastric resident balloons sealed with oNB-PAAM gel pin (top) shows no filler leakage in response to cyclic compression forces up to 10 N (middle). This resistance to cyclic compression stays the same if the gel is left undegraded for 24 hours (bottom). Photo Credit: Ritu Raman, MIT. (**b**) Top: Degradation of central portion of oNB-PAAM gel pin using 3 LED array (365 nm). Image shows LED array at a distance from balloon for clarity, but tests were carried out with LEDs directly above the gel to maximize light exposure intensity. Bottom: oNB-PAAM gel pins demonstrate color change in degraded region of the gel. Photo Credit: Ritu Raman, MIT. (**c**) Compression test of gastric resident balloons after oNB-PAAM gel pins have been degraded (top) shows filler leakage (right) under loads of 3 N, commensurate with peristaltic forces in the gut. Photo Credit: Ritu Raman, MIT.

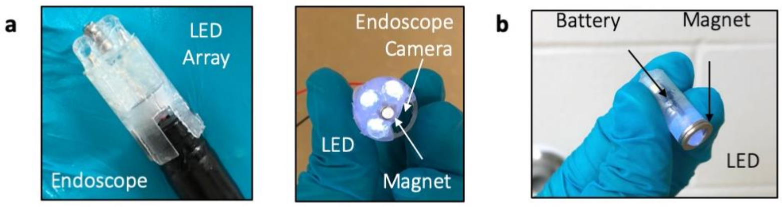


**Fig. S7. Custom-manufactured light-emitting devices for in vivo triggering of light-degradable hydrogels.** (**a**) Left: Photograph of LED array cap on endoscope. Right: Top view of endoscopic LED array with 3 LEDs turned on. Central magnet enables *in vivo* docking with the gastric-resident balloon and a hole in the cap enables retention of vision through the endoscope's on-board camera. Photo Credit: Ritu Raman, MIT. (**b**) Photograph of ingestible LED with light turned on. Photo Credit: Ritu Raman, MIT.

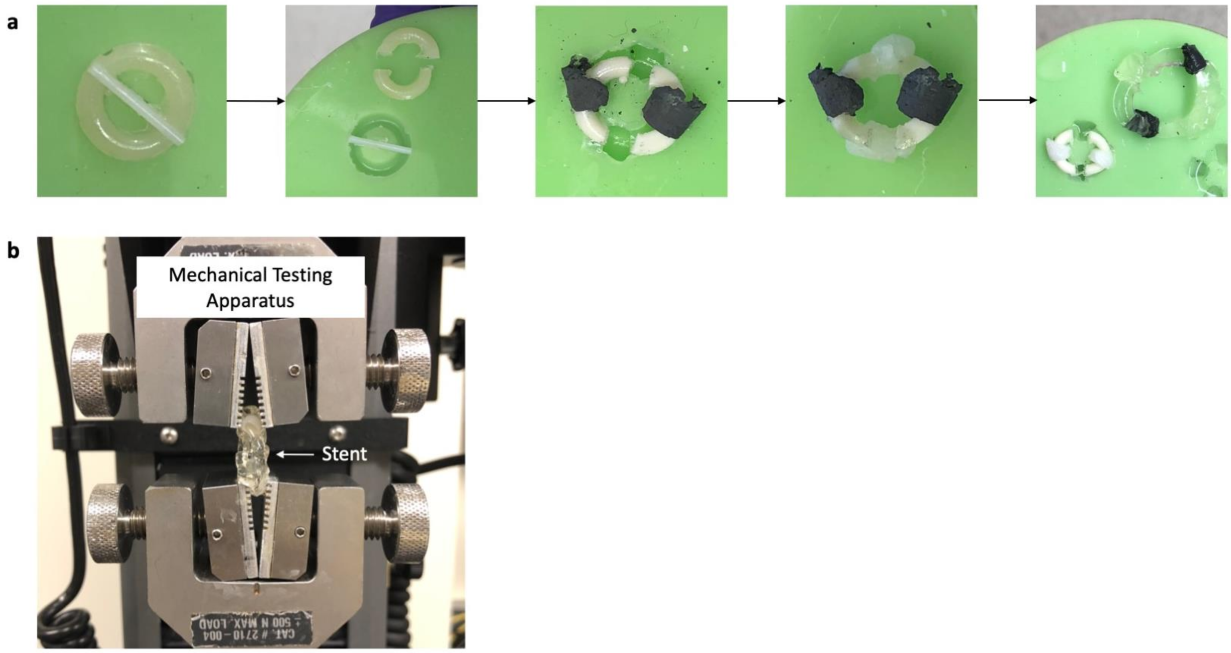


**Fig. S8. Synthesis and characterization of light-triggerable esophageal stent.** (**a**) Schematic of assembly process for esophageal stents showing, in sequence from left to right: casting two halves of oNB-PAMPS stent, removing from mold to dry, threading dried gel through PCL beads, sealing two halves together into complete stent, swelling to show size difference after immersion in liquid. Photo Credit: Ritu Raman, MIT. (**b**) Picture of *in vitro* compression testing of esophageal stents. Photo Credit: Ritu Raman, MIT.
